# Identification and Imaging of Prostaglandin Isomers Utilizing MS^3^ Product Ions and Silver Cationization

**DOI:** 10.1021/jasms.3c00233

**Published:** 2023-08-17

**Authors:** Leonidas Mavroudakis, Ingela Lanekoff

**Affiliations:** Department of Chemistry−BMC, Uppsala University, Uppsala 75123, Sweden

## Abstract

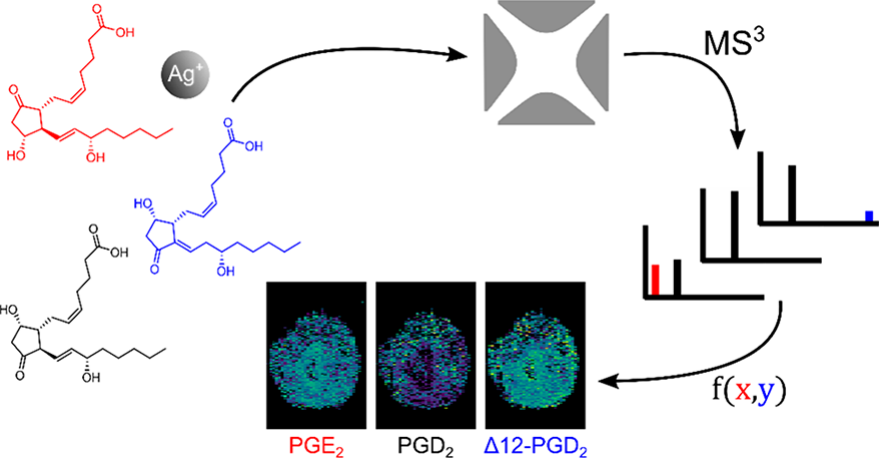

Prostaglandins (PGs) are important lipid mediators involved in physiological processes, such as inflammation and pregnancy. The pleiotropic effects of the PG isomers and their differential expression from cell types impose the necessity for studying individual isomers locally in tissue to understand the molecular mechanisms. Currently, mass spectrometry (MS)-based analytical workflows for determining the PG isomers typically require homogenization of the sample and a separation method, which results in a loss of spatial information. Here, we describe a method exploiting the cationization of PGs with silver ions for enhanced sensitivity and tandem MS to distinguish the biologically relevant PG isomers PGE_2_, PGD_2_, and Δ12-PGD_2_. The developed method utilizes characteristic product ions in MS^3^ for training prediction models and is compatible with direct infusion approaches. We discuss insights into the fragmentation pathways of Ag^+^ cationized PGs during collision-induced dissociation and demonstrate the high accuracy and robustness of the model to predict isomeric compositions of PGs. The developed method is applied to mass spectrometry imaging (MSI) of mouse uterus implantation sites using silver-doped pneumatically assisted nanospray desorption electrospray ionization and indicates localization to the antimesometrial pole and the luminal epithelium of all isomers with different abundances. Overall, we demonstrate, for the first time, isomeric imaging of major PG isomers with a simple method that is compatible with liquid-based extraction MSI methods.

## Introduction

Prostaglandins (PGs) are eicosanoids that are of utmost importance in various biological processes including inflammation, regulation of immune response, vascular permeability, and pregnancy.^1−3^ All PGs are derived from the oxidation of arachidonic acid (AA) through the action of cyclooxygenases (COX). Specifically, COX-1 and COX-2 oxidize AA released from phospholipids through the action of phospholipases to prostaglandin G_2_ (PGG_2_). The PGG_2_ then undergoes peroxidation to yield PGH_2_, which is highly unstable and is rapidly converted to PGD_2_, PGF_2a_, PGE_2_, PGI_2_, or thromboxane A_2_ (TXA_2_) by specific synthases.^1^ Both TXA_2_ and PGI_2_ are unstable and can be quickly hydrated in an aqueous solution.^2^ PGD_2_ can eventually degrade to PGJ_2_ through the intermediate Δ12-PGD_2_.^4,5^ The effects of individual prostaglandins are still largely debated since they possess pleiotropic effects, acting as both pro-inflammatory and anti-inflammatory mediators.^2^ For example, the positional isomers PGE_2_ and PGD_2_ both play crucial roles in inflammation. Recently, it has been argued that PGE_2_ and PGD_2_ exhibit opposing effects due to biased activities of their receptors,^6^ and it has been shown that their individual abundances can affect the severity of the disease.^7^ Additional studies have demonstrated the opposing effects of PGE_2_ and PGD_2_ in regulation of food intake, body temperature, sleep, and ischemic injury.^8−10^ Thus, to understand their individual role in biological systems, it is crucial to analytically identify the individual isomeric species.

Several analytical methods have been developed for the analysis of PGs, including enzyme immunoassays,^11−13^ liquid chromatography–mass spectrometry (LC-MS),^14−17^ supercritical fluid chromatography (SFC) MS,^18,19^ and gas chromatography–mass spectrometry (GC-MS).^20−22^ However, pinpointing PGs with isomeric resolution is not straightforward due to the presence of several isomers. For example, PGD_2_, PGE_2_, PGI_2_, Δ12-PGD_2_, TXA_2_, lipoxin A_4_ (LXA_4_), and LXB_4_ are all isomers with the same chemical formula; C_20_H_32_O_5_. Furthermore, additional isomers can be derived through nonenzymatic oxidation of AA to generate isoprostanes (iso-PG).^23^ Although immunoassays are attractive for analysis of PGs, since no expensive instrumentation is required, they can lack specificity resulting in recognition of additional PGs.^13^ The most common method is using chromatography in combination with mass spectrometry for bulk analysis of PGs, since preconcentration steps are important for detectability of the low-abundant endogenous prostaglandins. However, the combination of ion mobility spectrometry (IMS) and tandem MS can differentiate between different PG isomers without the time-consuming step of chromatography.^24,25^ Overall, all methods require homogenization of the sample prior analysis to remove the information about spatial distribution.

Spatial information on PG distribution can reveal insights into their action mechanism since PGs are mainly acting close to their production site.^1^ The various PGs bind to different receptors which are expressed by different types of cells, and thus, the cellular environment dictates the mixture of PGs that is present. For example, PGD_2_ is produced in high amounts from mast cells while PGE_2_ is produced mainly from monocytes and macrophages.^1^ Therefore, a spatially resolved analysis can provide further information on biological mechanisms and events. Mass spectrometry imaging (MSI) provides information on the spatial distribution of endogenous molecules directly from thin tissue sections.^26,27^ However, the detection of low-abundance species is challenging due to the lack of preseparation and/or preconcentration steps with this direct infusion approach. To overcome the limitation of low ionization efficiency and low abundance of PGs, Duncan et al. utilized the cationization of PGs with silver ions (Ag^+^) and demonstrated up to 30-fold increase in sensitivity, compared to the conventional analysis of PGs as deprotonated ions.^28^ Additionally, cationization with Ag^+^ or divalent metal cations and tandem mass spectrometry (MS^n^) can provide structural information on the isomeric composition of fatty acids, sphingolipids, and phospholipids,^29−35^ opening a new avenue also for analysis of prostaglandin isomers.

In this work, we leverage the increased sensitivity obtained when prostaglandins are cationized with silver ions and report characteristic product ions observed in MS^3^ that enable identification of the biologically relevant PG isomers; PGE_2_, PGD_2_, and Δ12-PGD_2_. Furthermore, we develop a prediction model based on the relative abundance of MS^3^ product ions and validate it using both standard solutions and complex samples. The developed model is used to predict the isomeric distribution of PGE_2_, PGD_2_, and Δ12-PGD_2_ in a mouse uterus implantation site analyzed by silver-doped pneumatically assisted nanospray desorption electrospray ionization (PA nano-DESI) MSI. Here, we show, for the first time, isomeric differentiation and distribution of major PG species directly from tissue sections using MSI.

## Materials and Methods

### Chemicals

Methanol, acetonitrile, isopropanol (all LC-MS grade), formic acid (98–100%), and silver nitrate (AgNO_3_, >99.8%) were purchased from Merck (Darmstadt, Germany). Prostaglandin standards (PGE_2_, PGD_2_, Δ12-PGD_2_, 8iso-PGE_2_, 15(R)-PGE_2_, ent-PGE_2_, 11β-PGE_2_, 5-trans-PGE_2_, 15(R)-PGD_2_, PGD_2_-d_9_, and PGF_2a_-d_9_) were purchased from Cayman Chemicals (MI, USA). Octadecanoyl (18,18,18–d_3_)-l-carnitine-HCl (C18-carnitine-d_3_), arachidonic acid-d_8_, and 10(Z),13(Z)-monononadecadienoin (MG 19:2) were purchased from Larodan AB (Solna, Sweden). Lysophosphatidylcholine 19:0 (LPC 19:0), phosphatidylcholine 11:0/11:0 (PC 11:0/11:0), and oleic acid-d_9_ were purchased from Merck. Monoisotopic silver (^107^Ag) was purchased from Trace Sciences International (Richmond Hill, ON, Canada), and a solution was prepared previously in-house.^33^ Deionized water was obtained from an in-house purification system (Millipore, Merck).

### Biological Samples

Flash frozen brain from healthy rats (Sprague–Dawley) was purchased from Innovative Research Inc. (Novi, MI, USA) and was used for creating a rat brain extract (RBE) that served as a complex sample (see Supporting Information for details). Thin tissue sections on regular glass slides from uterine implantation sites were obtained from *Trp53*^*d/d*^ mice on the eighth day of pregnancy and used after longer time storage in −80 °C freezer.^36^

### Training and Validation of Prediction Model

For model training and testing, standard solutions of PGE_2_, PGD_2_, and Δ12-PGD_2_, at various proportions (Table S1), were prepared and analyzed using flow injection analysis (FIA). The total PG concentration (PGE_2_ + PGD_2_ + Δ12-PGD_2_) was 3 μM in each solution containing 10 ppm of ^107^Ag^+^ and 0.1% formic acid in 9:1 methanol/acetonitrile (v/v). For FIA, the carrier solvent was methanol/water 9:1 (v/v) with 0.1% formic acid delivered at flow rate of 5 μL min^–1^, and 20 μL of each sample was injected three times (technical replicates) using a quaternary pump and autosampler maintained at 8 °C (Vanquish UHPLC, Thermo Fisher Scientific). The pump and autosampler modules were connected to an Orbitrap IQ-X tribrid mass spectrometer (Thermo Fisher Scientific, San Jose, CA, USA) for acquiring full MS (*m*/*z* 200–1500), selected ion monitoring (SIM) scans (*m*/*z* 445–485), and MS^3^ scans in both the orbitrap and ion trap (Table S2).

### LC-MS Analysis of PG Standards and RBE

Separation of PG isomers was achieved using either a short or longer gradient program (Tables S3 and S4) on a Kinetex C18 column (2.7 μm, 100 mm × 2.1 mm) maintained at 55 °C. The solvent flow rate delivered to the column was 0.4 mL min^–1^ through the Vanquish UHPLC system. For the analysis of neat PG standards, 5 μL of standard solution (2.8 μM in 30% ACN) were injected while for the analysis of rat brain extract, 10 μL (reconstituted in 30% acetonitrile (ACN), without preconcentration or dilution) were injected. An autosampler maintained at 8 °C was used to inject the samples. To facilitate formation of Ag^+^ adducts with PGs, 1000 ppm of AgNO_3_ at a flow rate of 4 μL min^–1^ was added post-column through a three-way tee resulting in a final concentration of 10 ppm Ag^+^. The LC method was coupled to an Orbitrap Velos Pro mass spectrometer that recorded a full MS in the orbitrap and MS^2^ in the orbitrap or MS^3^ in the ion trap, with parameters shown in Table S5.

### PA nano-DESI MSI of Mouse Uterus Implantation Site

The PA nano-DESI probe was constructed according to Duncan et al.^37^ with two 50/150 μm ID/OD fused silica capillaries (Polymicro Technologies, LLC., Phoenix, USA) as the primary and secondary capillary. The solvent was delivered at 0.5 μL min^–1^ using a syringe pump (Legato, KD Scientific) and contained 0.5 μM PGD_2_-d_9_, 0.5 μM PGF2a-d_9_, 0.5 μM C18-carnitine-d_3_, 2 μM arachidonic acid-d_8_, 2 μM oleic acid-d_9_, 0.5 μM MG 19:2, 1 μM LPC 19:0, 1 μM PC 11:0/11:0 in 9:1 acetonitrile/methanol v/v with 0.1% formic acid. The nitrogen gas flow for the nebulizer was adjusted until a stable liquid bridge was established (4.5 bar backpressure). The sample was moved under the PA nano-DESI probe at 20 μm s^–1^ along the *x*-axis and at steps of 100 μm for oversampling^38^ across the *y*-axis using motorized X-Y-Z stages (Newport, CA, USA) controlled by a custom-made program in LabVIEW.^39^ MSI Data were acquired using an Orbitrap IQ-X mass spectrometer with a method similar to the one used for training the prediction model (Table S6). The final pixel size in the acquired ion images was 31 × 100 μm^2^, based on the duty cycle of the mass spectrometer (1.55 Hz), the *x*-axis sample movement (0.02 mm sec^–1^), and the *y*-axis sample movement (100 μm). Note that the pixel size does not equal spatial resolution, which is hard to measure in the scanning direction (*x*) and limited by the movement in the stepping direction (*y*). When using a chemical gradient over a cellular region to provide a rough estimate we find the upper limit of the spatial resolution across the scanning direction of the PA nano-DESI probe to be 70 μm^40,41^ (Figure S1).

### Data Processing

Chemical structures were drawn using ChemDraw 18.1 (PerkinElmer Informatics Inc.). MS^3^ spectra and LC-MS data were visualized using Thermo Scientific FreeStyle (Thermo Fisher Scientific Inc.). Thermo RAW data were converted to centroided mzML using MSConvert^42^ (ProteoWizard) for subsequent processing in MATLAB R2022b (Mathworks, USA) using in-house developed scripts.^43^ For FIA analysis, 2.3 min of electrospray signal was averaged, resulting in ∼120 averaged scans. In all MS experiments, signals of interest were extracted from the .mzML data using a list of target *m*/*z* values. For orbitrap scan events, a mass tolerance of 5 ppm was used, while for ion trap scans the mass tolerance was 0.4 amu. Fitting of second degree polynomial surface and third degree polynomial curve on the training data set was done using MATLAB where the intensity of each product ion was divided by the sum of all product ions used to obtain the relative abundance. Cross-validation of the trained model was done using the hold-out method with 10% of the data set used for testing. Prediction of relative isomer abundances from PA nano-DESI MSI data was restricted to the [−10, 110]% range, and values outside that range were removed. The ion images only show signals from the tissue, since a mask based on the abundance of the MS^3^ product ion *m*/*z* 333.2 (intensity threshold = 30) was used. Further, a signal-to-noise (S/N) cutoff threshold was used for each product ion for inclusion in the model predictions (see Supporting Information for details). For region of interest (ROI) analysis of MSI data, the antimesometrial (AM) and mesometrial (M) pole regions were identified based on previous publication^36^ and data were extracted using in-house MATLAB scripts.^43^

## Results and Discussion

### Identification of Relevant Isomers

**Figure 1:**
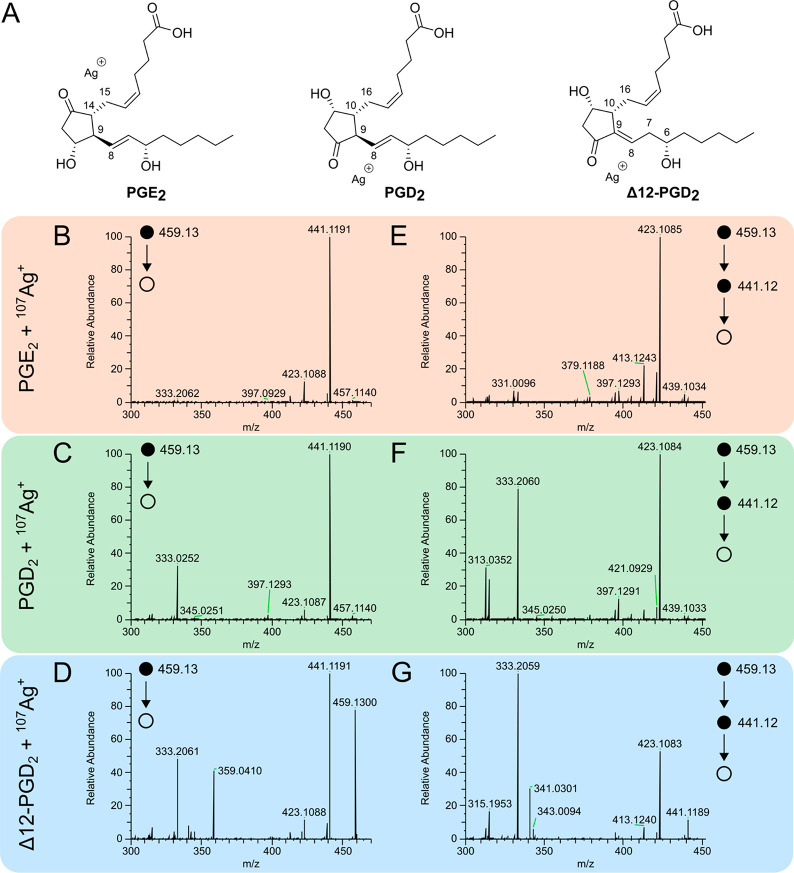
Structures (A) and obtained MS^2^ and MS^3^ spectra of PGE_2_, PGD_2_, and Δ12-PGD_2_ standards (B–G). (B–D) MS^2^ spectra from PGE_2_, PGD_2_, and Δ12-PGD_2_, respectively, when the molecular ion at *m*/*z* 459.13 was isolated and subjected to CID. (E–G) MS^3^ spectra of PGE_2_, PGD_2_, and Δ12-PGD_2_, respectively, after further CID fragmentation of the water-loss product ion at *m*/*z* 441.12.

It is well-known that several prostaglandin isomers can be present with the chemical formula C_20_H_32_O_5_. Specifically, the mass channel for PGE_2_ at *m*/*z* 459.1295 (M + ^107^Ag^+^) could potentially include eight biologically relevant isomers: PGE_2_, PGD_2_, Δ12-PGD_2_, LXA_4_, LXB_4_, TXA_2_, PGH_2_, and PGI_2_.^1^ The TXA_2_, PGH_2_, and PGI_2_ have been reported as highly unstable and therefore not likely to be detected.^1,2^ Additionally, lipoxins (LXA_4_ and LXB_4_) are rapidly metabolized *in vivo* through dehydrogenation and possible subsequent oxidation.^44^ However, the locations of the individual isomers have not been previously determined despite the importance of PGs in mouse embryo implantation.^28,36,45^ Therefore, the focus of this study is to separate the isomers PGE_2_, PGD_2_, and Δ12-PGD_2_ using mass spectrometry alone to facilitate MSI studies (Figure 1A).

### Tandem MS of Prostaglandin Isomers with Silver

Tandem MS provides a valuable tool for the structural identification of analytes based on their product ions. However, fragmentation of deprotonated prostaglandins in the negative ion mode mainly generate product ions resulting from water loss or other nonspecific losses of CO_2_ and C_6_H_12_O (Figure S2).^46^ In positive ion mode, only Δ12-PGD_2_ was detected as a protonated molecular ion, while all three standards were detected as sodiated molecular ions. For the protonated adduct, extensive loss of water was observed for all three isomers in MS^1^ due to source fragmentation (Figure S3). For the sodiated adducts, the major product ions in MS^2^ corresponded to a loss of water for all three isomers (Figure S2). In MS^3^, all sodiated isomer adducts showed product ions based on carbon chain losses in MS^3^ (Figure S2). Unfortunately, the loss of C_7_H_11_O_2_ was detected only with appreciable intensity in PGE_2_.

To increase sensitivity, as previously reported,^28^ monoisotopic silver ions ^107^Ag^+^ were included in the PA nano-DESI solvent. The use of monoisotopic silver reduces spectral overlaps and increases sensitivity as the signal is not diluted into two mass channels.^47^ As a result, silver cationized molecular ions of PGE_2_, PGD_2_, and Δ12-PGD_2_ were detected with high intensities in MS^1^ without any spontaneous loss of water (Figure S4). The MS^2^ product ions of PGE_2_, PGD_2_, and Δ12-PGD_2_ cationized with ^107^Ag^+^ mainly showed loss of water, especially for PGE_2_ (*m*/*z* 441.1191 and *m*/*z* 423.1088) (Figure 1B–D, Table 1). Contrarily, PGD_2_ gave rise to a product ion at *m*/*z* 333.0252, corresponding to the loss of C_8_H_16_O, and Δ12-PGD_2_ yields product ions with *m*/*z* 359.0410 (loss of C_6_H_12_O) and *m*/*z* 333.2061 (loss of AgH + H_2_O) (Table 1). To evaluate whether this product ion is unique for PGD_2_, a complex sample (rat brain extract) was analyzed with LC-MS to separate PGs from other potential interferences appearing at the same mass channel (isomers and/or isobars). However, the product ion *m*/*z* 333.0252 was also detected from other molecules upon LC-MS of a chemically complex biological sample (Figure S5). Thus, MS^2^ does not provide enough selectivity to distinguish the silver cationized isomers with mass spectrometry alone.

**Table 1:** Summary of Identified Product Ions from the MS^2^ and MS^3^ Spectra of PGE_2_, PGD_2_, and Δ12-PGD_2_ Standards Cationized with ^107^Ag^+^

				**Detected**^a^^,^^b^**in:**		
Product ion *m*/*z*	Assigned chemical formula	Mass error (ppm)	Loss from precursor C_20_H_32_O_5_^107^Ag	PGE_2_	PGD_2_	Δ12-PGD_2_
313.0352	C_13_H_18_O_2_^107^Ag	0.07	C_7_H_12_O_2_ + H_2_O	No	MS^3^ C10–C16	MS^3^ C10–C16
315.1953	C_20_H_27_O_3_	–0.45	H_2_O + H_2_O + ^107^AgH	No	MS^3^	MS^2^ & MS^3^
331.0096	C_12_H_16_O_4_^107^Ag	0.6	C_8_H_16_O	MS^3^ C8–C9	No	No
331.1905^c^	C_20_H_27_O_4_	0.33	H_2_O + ^107^AgH + H_2_	MS^2^ & MS^3^	MS^2^ & MS^3^	MS^2^ & MS^3^
333.0252	C_12_H_18_O_4_^107^Ag	0.12	C_8_H_14_O	No	MS^2^ C8–C9	No
333.206	C_20_H_29_O_4_	–0.03	H_2_O + ^107^AgH	MS^3^	MS^2^ & MS^3^	MS^2^ & MS^3^
341.0301	C_14_H_18_O_3_^107^Ag	–0.23	C_6_H_12_O + H_2_O	No	No	MS^2^ & MS^3^ C6–C7
343.0094	C_13_H_16_O_4_^107^Ag	–0.06	C_7_H_16_O	No	No	MS^3^ C7–C8
359.041	C_14_H_20_O_4_^107^Ag	0.95	C_6_H_12_O	No	No	MS^2^ C6–C7
397.1291	C_19_H_30_O_2_^107^Ag	0.3	CH_2_O_3_ (CO_2_ + H_2_O)	MS^3^	MS^3^	No
413.1245	C_19_H_30_O_3_^107^Ag	–0.11	CH_2_O_2_	MS^3^	MS^3^	MS^3^
423.1084	C_20_H_28_O_3_^107^Ag	0.02	H_2_O + H_2_O	MS^2^ & MS^3^	MS^2^ & MS^3^	MS^2^ & MS^3^
441.119	C_20_H_30_O_4_^107^Ag	–0.06	H_2_O	MS^2^	MS^2^	MS^2^ & MS^3^

a Detected at >5% relative abundance to the base peak.

b Where applicable, the MS^n^ level is noted as well as the C–C cleavage responsible (see Figure 1A).

c Detected with relative abundance below 5%, but it is mentioned here due to the possible isobaric interference to 331.0096.

### Identification of Characteristic Product Ions in MS^3^

To increase selectivity, MS^3^ was performed with silver cationized PG isomers PGE_2_, PGD_2_, and Δ12-PGD_2_ after neutral loss of water in MS^2^ (Figure 1E–G). A product ion of PGE_2_ was observed at *m*/*z* 331.0096, which was determined to originate from the cleavage of C8–C9 in PGE_2_ based on accurate mass (Table 1, Figures 1A,E and S6). This product ion is an important marker for the presence of PGE_2_ since it was only detected below 2% relative intensity in any of the other two PG isomers. Similarly, the MS^3^ product ion at *m*/*z* 341.0301 was characteristic of Δ12-PGD_2_ (Table 1), and it formed after cleavage of the bond between C6–C7 of Δ12-PGD_2_ (Figure 1A,G). All three isomers gave the product ion with *m*/*z* of 333.2060 in MS^3^ (Table 1, Figure 1E–G). However, despite this product ion occurring after noncharacteristic losses of water and AgH from all three isomer precursors, which has been previously described,^30,31,33,48−51^ the abundance of *m*/*z* 333.2060 is highly dependent on the isomeric structure.

The different abundances of the detected product ion after H_2_O and AgH (*m*/*z* = 333.2060) loss are found to be highly specific for each isomer (Table 1, Figure 1E–G). Specifically, the PGE_2_ isomer produces this product ion at 5% relative abundance (relative to the base peak; Figure 1E). Contrarily, the two PGD_2_-type structures, PGD_2_ and Δ12-PGD_2_, show much higher intensities of the product ion *m*/*z* 333.2060, about 80% and 100%, respectively. This increased formation of this product ion is related to the position of the carbonyl group in the ring of PGD_2_ and Δ12-PGD_2_ that is involved in stabilizing the product ion (Figure S7). This was validated using PGF2a-d_9_ that does not contain carbonyl groups in the ring and only formed the product ion *m*/*z* 333.2060 with extremely low intensity (Figures S7 and S8). Therefore, the presence of the carbonyl group is involved in the abstraction of an alpha-hydrogen by the silver ions, making it important for the formation of this product ion and thereby useful for distinguishing the isomers. Previous work studying the interactions of metal ions with phospholipids revealed that the carbonyl moieties and the double rings were important for the interaction of the metal ions with the analytes.^33,52,53^ Collectively, these observations and our data suggest that the silver ions mainly interact with the carbonyl group in the ring and the adjacent double bond (Figure 1A).

### Validation of MS^3^ Product Ions

To identify the distinct isomer species based on product ions, it is important that the product ions a) differ in abundance among the three isomers and b) do not arise from other molecules of the complex sample. Although the product ion at *m*/*z* 333.2060 is common for all three isomers, it does not originate from other molecules, as shown with our LC-MS analysis of rat brain extract (Figure S9). Additionally, based on the LC-MS^n^ measurements, the product ions with *m*/*z* 331.0096 and 341.0301 were not found to be originating from other molecules than PGs (Figure S9). However, despite the fact that the isomers 15(R)-PGE_2_, 15(R)-PGD_2_, ent-PGE_2_, 8-iso-PGE_2_, 11β-PGE_2_, and 5-trans-PGE_2_ (Figure S10) produce the same product ions, it is well-known that the major isomers are PGE_2_ or PGD_2_, and therefore any potential contribution from other isomers should be minimal.^2^ Notably, we did observe that the relative abundance of *m*/*z* 331.0096 and 333.2060 was slightly different among PGE_2_, 8-iso-PGE_2_, and 11β-PGE_2_ but at abundances lower than 10% of the base peak in the MS^3^ spectrum (Figure S10). Thus, we find that the detected MS^3^ production ions at *m*/*z* 331.0096, 333.2060, and 341.0301 are specific to the three isomers PGE_2_, PGD_2_, and Δ12-PGD_2_.

### MS^3^ Using Ion Traps

**Figure 2:**
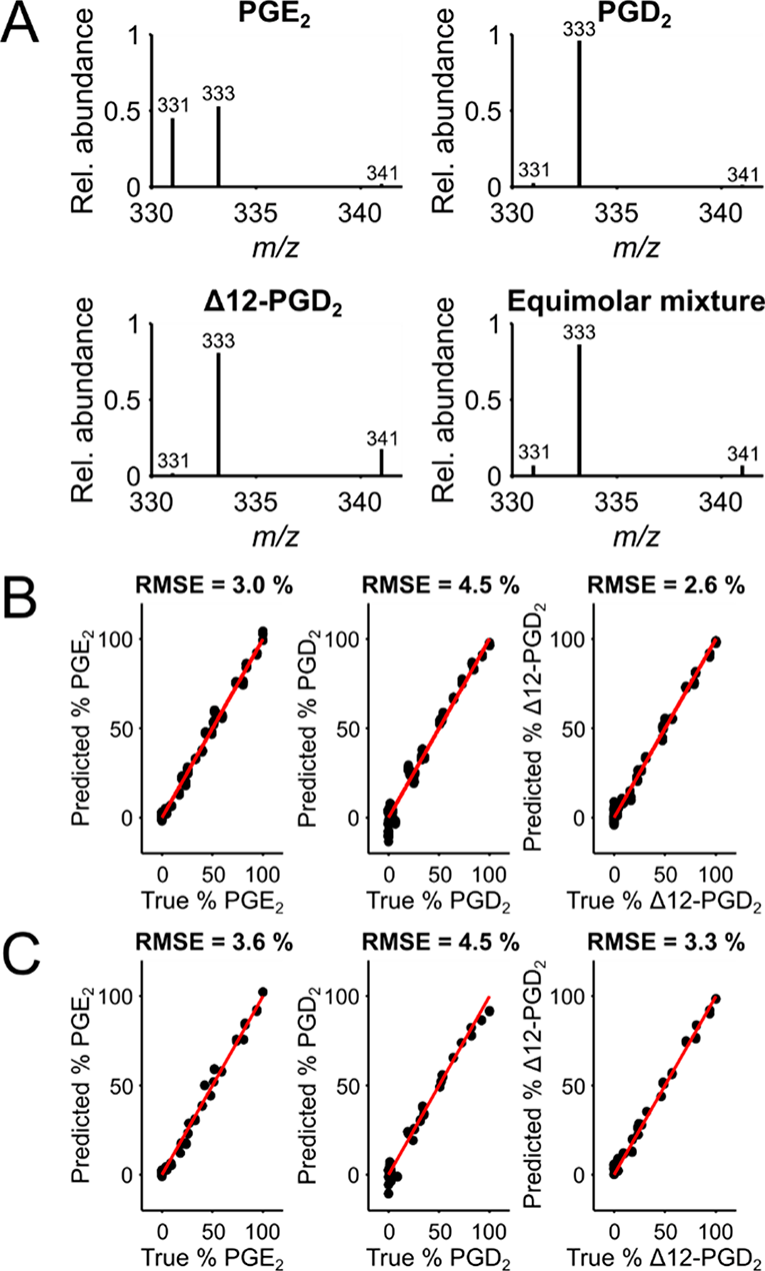
Modeling of MS^3^ product ions from PGE_2_, PGD_2_, and Δ12-PGD_2_. (A) Relative abundance (the intensity of each product ion is divided by the sum of all three product ions) of *m*/*z* 331, 333, and 341 from PGE_2_, PGD_2_, Δ12-PGD_2_ and an equimolar mixture of all three isomers. (B) Cross-validation of the model for predicting PGE_2_, PGD_2_, and Δ12-PGD_2_ using the “hold-out” method and 50 iterations. The average RMSE is shown for each prediction. (C) Evaluation of the model using a data set of complex samples spiked with PGE_2_, PGD_2_, and Δ12-PGD_2_ at various proportions. The RMSE is shown for each prediction.

The data for identifying product ions were generated by using high mass resolving power in the orbitrap. This allows for the necessary accurate mass measurements for formula assignments of neutral losses and product ions. However, the duty cycle for acquiring MS^3^ data using an orbitrap is quite long compared to an ion trap. The gain in speed improves the signal-to-noise ratio (S/N) and thereby the sensitivity by generating more data per time.^54^ However, the lower resolving power cannot resolve the two product ions originating from PGE_2_ in MS^3^, the unique *m*/*z* 331.0095 and the *m*/*z* 331.1905, where the *m*/*z* 331.1905 occurs by loss of H_2_ from the PGE_2_ product ion at *m*/*z* 333.2061. Nevertheless, due to the high correlation between the PGE_2_ production ions *m*/*z* 331.0095/333.2060 and 331.1905/333.2060, the interference of the signal from *m*/*z* 331.1905 in the ion trap data is not a problem (Figure S11). Thus, the lower-resolution scans can be utilized for data acquisition and to model the relative abundances of the three PG isomers in the mass channel. Overall, it is clear that the composition of isomers significantly alters the relative abundances of the three product ions, 331, 333, and 341, which is key for developing a predictive method (Figure 2A).

### Modeling of MS^3^ Product Ions

Diagnostic product ions are ideal for reliable identification of molecular species and isomers in tandem MS, although the relative abundance of selected product ions can be used to predict the identity of molecular species and isomers.^33,34,55−57^ The selected MS^3^ product ions detected in the ion trap at *m*/*z* 331, 333, and 341 were used to create a model that would enable prediction of the PGE_2_, PGD_2_, and Δ12-PGD_2_ ratio. Specifically, standard solutions containing between 0 and 100% of PGE_2_, PGD_2_, and Δ12-PGD_2_ in MS^3^ were analyzed, and the intensities of *m*/*z* 331, 333, and 341 in MS^3^ were extracted (Table S1, training data set). Subsequently, the relative abundance of each product ion was calculated and used for the model.

To generate the model that predicts the proportion of Δ12-PGD_2_ in each solution, the measured proportions were plotted against the acquired relative abundances of *m*/*z* 331 and 341. Following, the relative abundance of Δ12-PGD_2_ was modeled to a second degree polynomial surface and fitted (*R*^2^ = 0.994) through the data points (Figure S12, left) to obtain eq 1
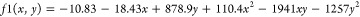
1where *x* is the relative abundance of *m*/*z* 331 and *y* the relative abundance *m*/*z* 341. The predicted % of Δ12-PGD_2_ is obtained by substituting the measured relative abundances of *m*/*z* 331 and *m*/*z* 341 in eq 1.

The relative abundance of the isomer PGE_2_ was modeled based on the relative abundance of the product ion with *m*/*z* 331 (Figure S12, right). A third-degree polynomial curve was fitted through the data (*R*^2^ = 0.988) providing eq 2
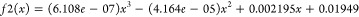
2where *x* is the predicted % PGE_2_. eq 2 can be solved numerically to obtain the predicted % PGE_2_ that is a real and positive number. The proportion of PGD_2_ in the sample is determined based on the predictions of the relative abundance of PGE_2_ and Δ12-PGD_2_ so that the total abundances sum up to 100.

### Validation of the Model

The cross-validation of the model was performed using the “Holdout” method where 10% of the data set (randomly selected) was kept for testing and the rest for training the model. The cross-validation was repeated 50 times, and the average root mean squared error (RMSE) was less than 5% for each isomer prediction (Figure 2B). The highest RMSE (4.5%) was found for PGD_2_, which is likely due to this value being dependent on the prediction of PGE_2_ and Δ12-PGD_2_ thereby accumulating the error. Note that the relative abundance of the product ions is dependent on the collision-induced dissociation (CID) settings and that the same settings need to be used for both model training and predictions (Figure S13). The model was further validated in a complex chemical matrix of molecules extracted from rat brain tissue by spiking different proportions of each isomer into the sample (Table S1). Despite the increased chemical complexity, the accuracy was high with an average RMSE below 5% for each isomer (Figure 2C). Thus, the model was found to be robust and accurate in predicting the relative abundance of PGE_2_, PGD_2_, and Δ12-PGD_2_.

### Imaging of Major PG Isomers in Tissue

**Figure 3:**
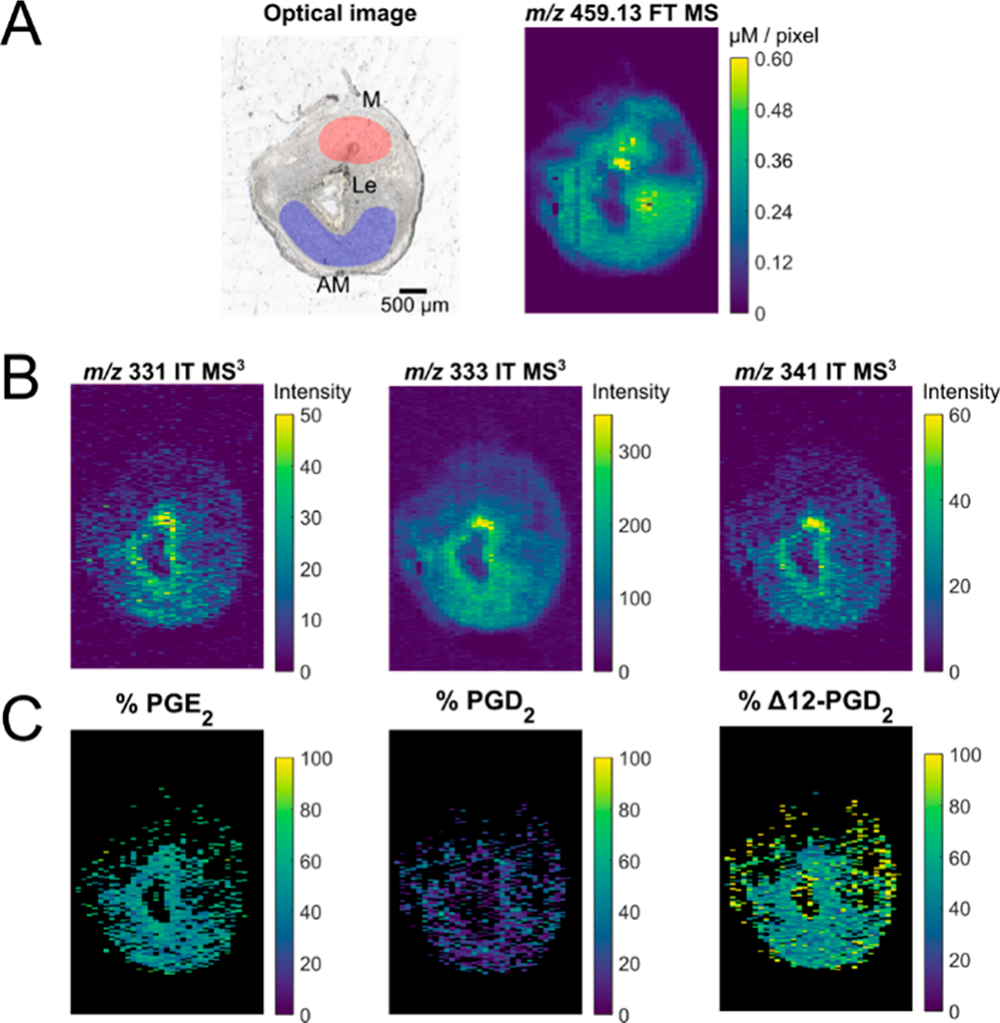
MSI of the three PG isomers. (A) Optical image of the analyzed mouse embryo implantation site where the antimesometrial (AM) pole, mesometrial (M) pole, and luminal epithelium (Le) are marked. Quantitative ion image of the precursor at *m*/*z* 459.13 ± 5 ppm acquired using SIM scan, normalized to the internal standard PGD_2_-d_9_ (0.5 μM) doped in the PA nano-DESI solvent and converted to detected concentration per pixel. (B) Raw intensities of MS^3^ product ions (459.13 → 441.12) measured in the ion trap (±0.4 amu). (C) Predicted relative abundances (%) of PGE_2_, PGD_2_ and Δ12-PGD_2_. Each isomer abundance is determined relative to the other so that all three isomers’ abundance in each pixel adds up to 100%. Pixels with black color gave predictions outside the 0–100% range and were set to 0.

We have previously reported imaging of PGs in mouse pregnancy models.^28,36^ However, in these studies, the identification of isomers was determined by LC-IMS-MS of homogenized tissue, which does not reveal potential differences in the cellular regions. Here, we apply our prediction model in a proof-of-principle study to identify PG isomers and their distributions directly in mouse embryo implantation sites. To gain information on both the precursor ion location and the isomers, high-resolution orbitrap scans in MS^1^ were interlaced with ion trap scans in MS^3^ during image acquisition. The quantitative ion image of the PG precursor ion at *m*/*z* 459.1295 (±5 ppm) shows the intricate distribution of PG over the tissue (Figure 3A). One-point quantitation was achieved in our i2i software using the ratio of the intensity of the endogenous *m*/*z* 459.1295 to the standard PGD_2_-d_9_ in each pixel multiplied by the concentration of the standard.^43,58^

The distributions of the selected MS^3^ product ions at *m*/*z* 331, 333, and 341 are seemingly similar (Figure 3B). Particularly, they clearly show a higher abundance in the antimesometrial (AM) pole and the luminal epithelium (Figure 3B). Noteworthy, the difference between the precursor and product ion images indicate that the precursor mass channel includes additional isomers or isobars that contribute to the observed image of *m*/*z* 459.13. The less uniform ion images of the product ions with *m*/*z* 331 and 341 are consistent with their lower intensity and yield (Figure 2A). To gain insight into the isomeric distributions, the intensities of the three selected MS^3^ product ions were extracted from each pixel of the ion image. Subsequently, the relative abundance of each isomer was determined using the developed prediction models in the order of PGE_2_ followed by Δ12-PGD_2_ and finally PGD_2_. A filtering based on S/N was applied to include only pixels with S/N above 5, thereby providing a higher confidence in the predictions (Figure S14) (see Supporting Information for details).

The resulting relative abundance of each isomer in the tissue portrays unique results with dynamic changes in the cellular regions (Figure 3C). Specifically, none of the isomers show localization to the mesometrial (M) pole, which is consistent with previous reports demonstrating lower abundance in this region.^36^ The PGE_2_ isomer and Δ12-PGD_2_ are detected mainly in the luminal epithelium. All isomers show the highest abundances in the AM-pole (Figure S15), although PGD_2_ is the least abundant of all three isomers. A region-of-interest (ROI) analysis of the AM-pole, selected as indicated in the optical image, shows that the isomeric composition of the detected PGs in the AM-pole is on average 35% (±23.7) PGE_2_, 15% (±16) PGD_2_, and 50% (±16) Δ12-PGD_2_. The larger deviation in the AM-pole for PGD_2_ is due to the lower abundance of the isomer in this region that increases the error in the prediction. Overall, the pixel-to-pixel standard deviation is much larger than the model prediction error (as indicated by the RMSE for each isomer in Figure 2), which implies that the biological variability is higher than the technical.

Previous reports have independently suggested that PGE_2_ and PGD_2_ are involved in embryo implantation.^36,59−61^ For the first time, we can show that both PGE_2_ and PGD_2_ are involved and that the localizations in the mouse embryo implantation site on day 8 of pregnancy are similar. With our method, we found that PGD_2_ is the least abundant out of the three investigated PG isomers. However, PGD_2_ degradation could have occurred during long time storage since both degradation products Δ12-PGD_2_ and Δ12-PGJ_2_ ([M+^107^Ag]^+^*m*/*z* 441.119) were detected (Figure S16).^4,5^ Overall, our prediction model enables generation of ion images and ROI analyses that have the potential to reveal intricate details of biological systems down to isomeric interactions.

## Conclusion

Determination of individual isomers is important for a deeper understanding of biological mechanisms in health and disease. In this work, we have developed a method to distinguish the major prostaglandin isomers PGE_2_, PGD_2_, and Δ12-PGD_2_ using tandem MS. The method utilizes differences in the MS^3^ spectra of prostaglandins when cationized with silver ions. A robust and highly accurate prediction model was trained to determine the abundance of each isomer in mixtures. The method is compatible with direct infusion approaches and was applied in mass spectrometry imaging of mouse uterus implantation sites at day 8 of pregnancy. We show for the first time imaging of prostaglandin isomers directly from a thin tissue section and reveal the individual localization and abundances of each isomer. Overall, this simple method is anticipated to contribute to further understanding of prostaglandins in inflammation and pregnancy.

## Supporting Information Available

Preparation of rat brain extract, Training and testing data sets for the prediction model, LC-MS gradients used, MS parameters, Calculations for spatial resolution, MS^n^ spectra of deprotonated and sodiated PGs, LC-MS of PG isomers, fragmentation pathways for PG isomers, MS^3^ of PGF_2a_-d_9_, comparison of FT and IT data, prediction models, MS^3^ of iso-PGs, MS^3^ of PGE_2_/PGD_2_ mixture with different CID level, S/N filtering for MSI data, RGB overlay of isomeric images, ion image of *m*/*z* 441.1190 (putative Δ12-PGJ_2_ + ^107^Ag^+^) (PDF)
