# Supplementary material for: Identification and Imaging of Prostaglandin Isomers Utilizing MS3 Product Ions and Silver Cationization
Source: J Am Soc Mass Spectrom. 2023 Aug 17;34(10):2341–9. doi: 10.1021/jasms.3c00233 (PMC10557378; doi:10.1021/jasms.3c00233)
Supplement: Supplementary file 1 — js3c00233_si_001.pdf [file js3c00233_si_001.pdf]

Supporting information

Identification and imaging of prostaglandin isomers utilizing MS^3 product ions and silver cationization

Leonidas Mavroudakis, Ingela Lanekoff*

Dept. of Chemistry – BMC, Uppsala University, Uppsala 75123, Sweden

*Corresponding author: ingela.lanekoff@kemi.uu.se

Preparation of rat brain extract (RBE). One intact brain from a Sprague Dawley rat (Innovative Research Inc., MI, US) weighing 1.17607 g was added to 33.976 mL methanol and sonicated (VCX 130, Sonics and Materials, Inc.) for 2 cycles (50% power for 5 min) using a pulse program consisting of 2 s pulses followed by 1 s pause. Subsequently, the homogenate was centrifuged at 2000g for 6 min, the supernatant was removed and stored in -20 °C until analysis.

Table S1. Samples analyzed for training and testing the model. The proportion of each isomer is determined as the amount of that isomer divided by the total amount of all three isomers. The total concentration of the three isomers was in every case 3 μM and all solutions contained 10 ppm $^{107}Ag^+$. The testing dataset contained also 20-times diluted rat brain extract.

	% PGE_2	% PGD_2	% Δ12-PGD_2
Training dataset	83.6	0.0	16.4
	52.1	0.0	47.9
	19.8	0.0	80.2
	33.3	35.6	31.2
	25.4	4.0	70.6
	23.4	72.9	3.6
	73.2	3.9	22.9
	23.5	54.1	22.4
	51.4	25.0	23.6
	25.7	26.0	48.3
	0.0	0.0	100.0
	93.9	2.7	3.4
	3.6	92.9	3.6
	4.6	1.6	93.8
	8.8	64.4	26.7
	9.4	33.6	56.9
	59.6	32.6	7.8
	39.8	51.4	8.8
	43.2	6.8	50.1
	0.0	100.0	0.0
	100.0	0.0	0.0
	0.0	21.4	78.6
	0.0	52.6	47.4
	0.0	84.4	15.6
	80.2	19.8	0.0
	49.3	50.7	0.0
	17.3	82.7	0.0
Testing dataset	82.3	0.0	17.7
	51.9	0.0	48.1
	19.2	0.0	80.8
	32.8	35.1	32.1
	26.7	2.6	70.7

	23.9	72.3	3.7
	73.7	2.7	23.6
	23.7	53.3	23.0
	51.3	24.3	24.5
	25.7	25.5	48.8
	0.0	0.0	100.0
	93.9	2.2	3.9
	4.0	92.0	3.9
	4.8	1.3	93.9
	8.8	64.1	27.1
	9.3	33.8	56.9
	59.0	31.5	9.5
	39.9	50.9	9.2
	42.3	8.7	49.0
	0.0	0.0	0.0
	0.0	100.0	0.0
	100.0	0.0	0.0
	0.0	19.7	80.3
	0.0	53.7	46.3
	0.0	82.0	18.0
	80.9	19.1	0.0
	48.0	52.0	0.0
	18.1	81.9	0.0

Table S2. Ion source and MS parameters for the analysis of standards shown in Table S1 using the FIA method and the Orbitrap IQ-X.

**Source type**	Heated ESI
**Ion transfer capillary temperature**	275 °C
**Source heater temperature**	0 °C
**Sheath gas flow (a.u.)**	2
**Auxiliary gas flow (a.u.)**	2
**Sweep gas flow (a.u.)**	0
**Polarity**	Positive
**Spray voltage**	3.5 kV
**S-lens RF level**	60 %
**Activation type**	CID
**Activation energy**	40 %
**Ion trap MS^n AGC target**	1e4
**Ion trap MS^n maximum injection time**	500 ms
**Ion trap MS^n microscans**	7
**Orbitrap Full MS AGC target**	2e5
**Orbitrap Full MS maximum injection time**	200 ms

**Orbitrap Full MS microscans**	1
**Orbitrap Full MS resolution**	120000
**Orbitrap SIM AGC target**	5e4
**Orbitrap SIM maximum injection time**	200 ms
**Orbitrap SIM microscans**	1
**Orbitrap SIM resolution**	120000
**Orbitrap MS^n AGC target**	5e4
**Orbitrap MS^n maximum injection time**	"Auto"
**Orbitrap MS^n microscans**	1
**Orbitrap MS^n resolution**	60000
**MSn isolation width (quadrupole isolation)**	1 amu

Table S3. Short gradient program for the separation of PGE_2 and PGD_2. Solvent A: Water with 0.1% formic acid, Solvent B: Acetonitrile with 0.1 % formic acid, Solvent C: Isopropanol

**Time (min)**	**% A**	**% B**	**% C**
0	70	30	0
5	60	40	0
5.01	2	98	0
10	2	98	0
10.01	0	2	98
14	0	2	98
14.01	70	30	0
19	70	30	0

Table S4. Long gradient program for separation of PGE_2, PGD_2 and Δ12-PGD_2. Solvent A: Water with 0.1% formic acid, Solvent B: Acetonitrile with 0.1 % formic acid, Solvent C: Isopropanol

**Time (min)**	**% A**	**% B**	**% C**
0	75	25	0
5	60	40	0
15	2	98	0
15.01	0	98	2
25	0	2	98
25.01	75	25	0
35	75	25	0

Table S5. Ion source and MS parameters for the analysis of standards or rat brain extract using the LC-MS method in the Orbitrap Velos Pro.

**Source type**	Heated ESI
**Ion transfer capillary temperature**	275 °C
**Source heater temperature**	300 °C
**Sheath gas flow (a.u.)**	35

**Auxiliary gas flow (a.u.)**	10
**Sweep gas flow (a.u.)**	10
**Polarity**	Positive
**Spray voltage**	3.5 kV
**S-lens RF level**	65 %
**Activation type**	CID
**Activation energy**	30 %
**Ion trap MS^n AGC target**	1e4
**Ion trap MS^n maximum injection time**	500 ms
**Ion trap MS^n microscans**	7
**Orbitrap Full MS AGC target**	2e5
**Orbitrap Full MS maximum injection time**	200 ms
**Orbitrap Full MS microscans**	1
**Orbitrap Full MS resolution**	120000
**Orbitrap SIM AGC target**	5e4
**Orbitrap SIM maximum injection time**	200 ms
**Orbitrap SIM microscans**	1
**Orbitrap SIM resolution**	120000
**Orbitrap MSn AGC target**	5e4

Table S6. Ion source and MS parameters for the PA nano-DESI MSI experiments using the IQ-X.

**Source type**	Nanospray ionization (NSI)
**Ion transfer capillary temperature**	300 °C
**Polarity**	Positive
**Spray voltage**	3.0 kV
**S-lens RF level**	60 %
**Activation type**	CID
**CID activation energy**	40 %
**Ion trap MSn AGC target**	3e4
**Ion trap MSn maximum injection time**	500 ms
**Ion trap MSn microscans**	7
**Orbitrap Full MS AGC target**	2e5
**Orbitrap Full MS maximum injection time**	200 ms
**Orbitrap Full MS microscans**	1
**Orbitrap Full MS resolution**	120000
**Orbitrap SIM AGC target**	5e4
**Orbitrap SIM maximum injection time**	200 ms
**Orbitrap SIM microscans**	1
**Orbitrap SIM resolution**	120000
**MSn isolation width (quadrupole isolation)**	1 amu

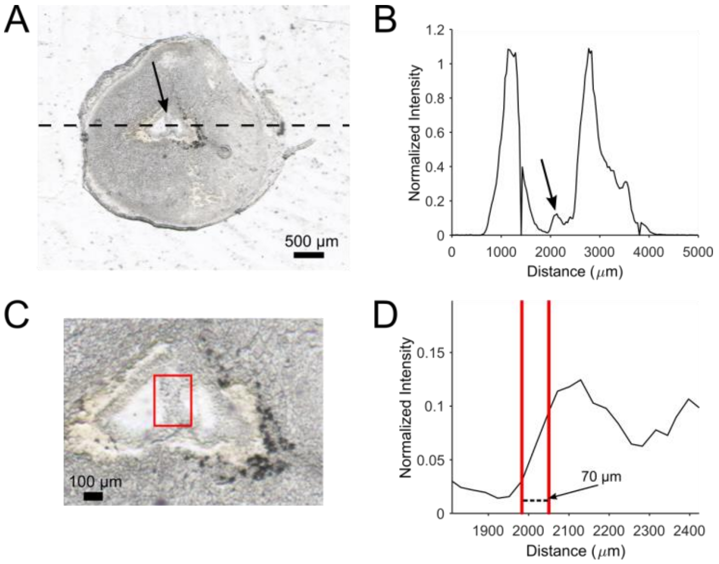

Figure S1. Estimation of spatial resolution in PA nano-DESI MSI experiment. A) Optical image of mouse uterus implantation site tissue section where the dashed line shows the line scan that was used for spatial resolution calculations. The arrow indicates a distinct morphological feature. B) Line scan of arachidonic acid normalized to arachidonic acid-d_8. The arrow indicates the morphological feature shown in A. C) Close up of the region of interest for spatial resolution calculations. D) Estimation of spatial resolution via the signal intensity change (from 20 % to 80 % of maximum). The same plot as in B is depicted.

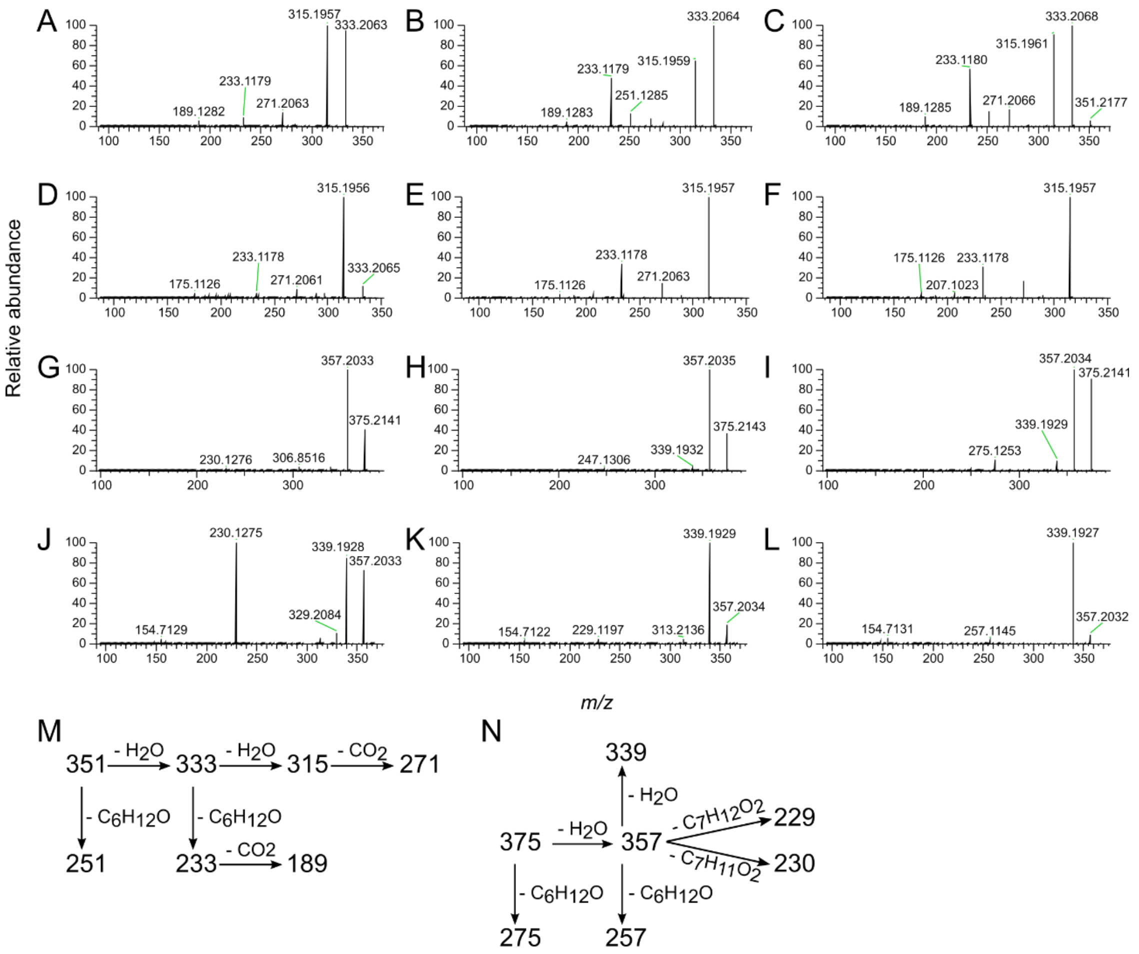

Figure S2. Tandem MS of deprotonated and sodiated PG isomers. A-C) MS^2 spectra of deprotonated PGE_2, PGD_2 and $\Delta 12$-PGD_2, respectively, detected at m/z 351.2176. D-F) MS^3 spectra of deprotonated PGE_2, PGD_2 and $\Delta 12$-PGD_2, respectively, for the transition 351.22 -> 333.21. G-I) MS^2 spectra of sodiated PGE_2, PGD_2 and $\Delta 12$-PGD_2, respectively, detected at m/z 375.2143. J-L) MS^3 spectra of sodiated PGE_2, PGD_2 and $\Delta 12$-PGD_2, respectively, for the transition 375.21 -> 357.20. M) Dissociation pathways for deprotonated PGs. N) Dissociation pathways for sodiated PGs.

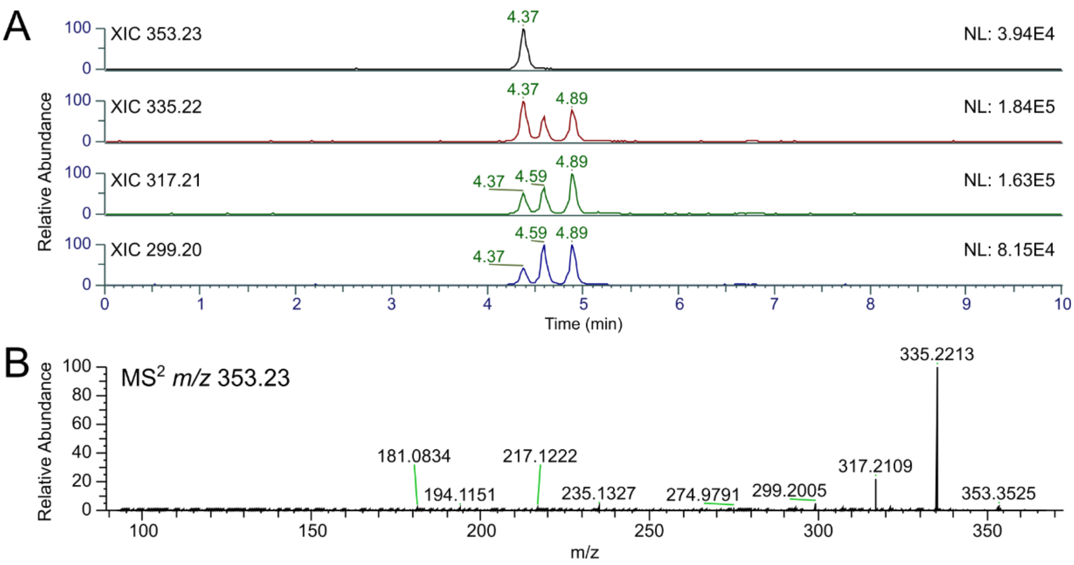

Figure S3. Protonated adducts of PGs in LC-MS and MS^2. A) Separation of Δ12-PGD$_2$, PGE$_2$ and PGD$_2$ standards (mentioned with their elution order) using LC-MS using a gradient starting with 25 % acetonitrile and 0.1 % formic acid – 75 % 0.1 % formic acid and increasing to 40 % acetonitrile over 5 minutes. The initial conditions are returned over the next 5 minutes. Shown are the XICs of protonated PGs at m/z 353.23 and water losses at m/z 335.22, 317.21 and 299.20. Only Δ12-PGD$_2$ is detected as intact protonated adduct while all three isomers show extensive loss of water. B) MS^2 spectrum of m/z 353.23 corresponding to protonated Δ12-PGD$_2$ where mainly water losses are detected.

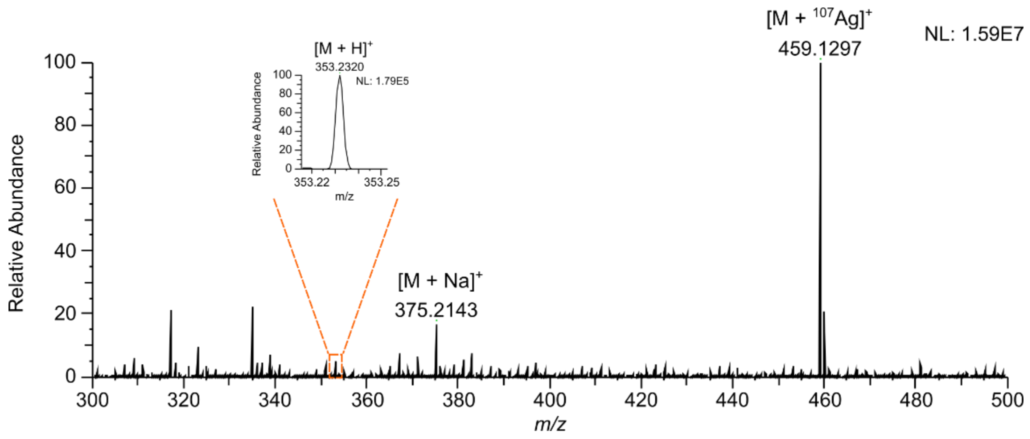

Figure S4. Full MS spectrum of a solution containing equal concentrations of PGE$_2$, PGD$_2$ and Δ12-PGD$_2$. The solvent contained 10 ppm $^{107}Ag^+$, 0.1 % formic acid in 9:1 v/v methanol:acetonitrile. The silver adduct at m/z 459.1297 is observed with ~5-fold larger intensity than the sodiated adduct at m/z 375.2143 and ~100-fold difference compared to the protonated adduct at m/z 353.2320.

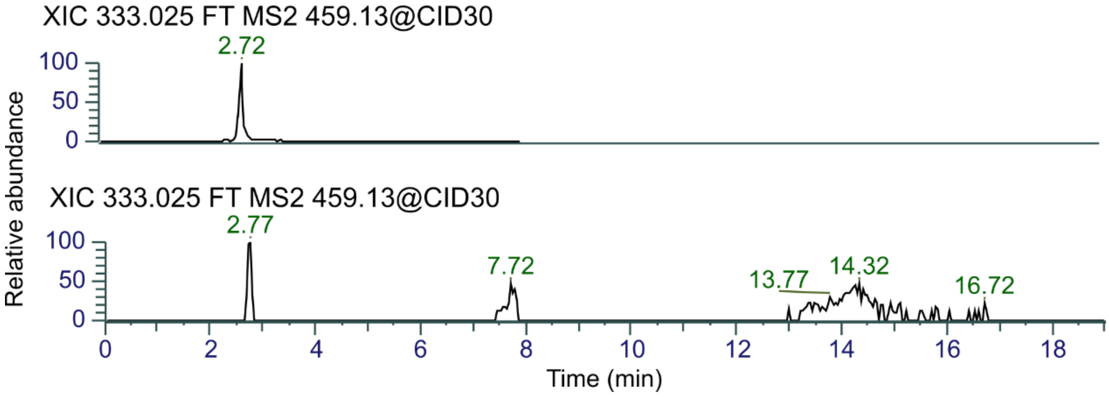

Figure S5. LC-MS analysis of PGE_2 and PGD_2 standards mixture and of complex sample (rat brain extract) with post-column addition of $AgNO_3$. Top: Injection of PGE_2 and PGD_2 standard solution (2.8 μM each). PGD_2 elutes at 2.72 min based on the XIC of product ion m/z 333.025 which is not produced from PGE_2. A shorter gradient was used compared to the bottom panel. Bottom: Injection of rat brain extract. Data were acquired using a method with FT-MS and FT-MS^2 (precursor: 459.13) scan events. In both panels, the XIC for the product ion with m/z 333.025 in the MS^2 spectrum is shown. The gradient program shown in Table S2 was used.

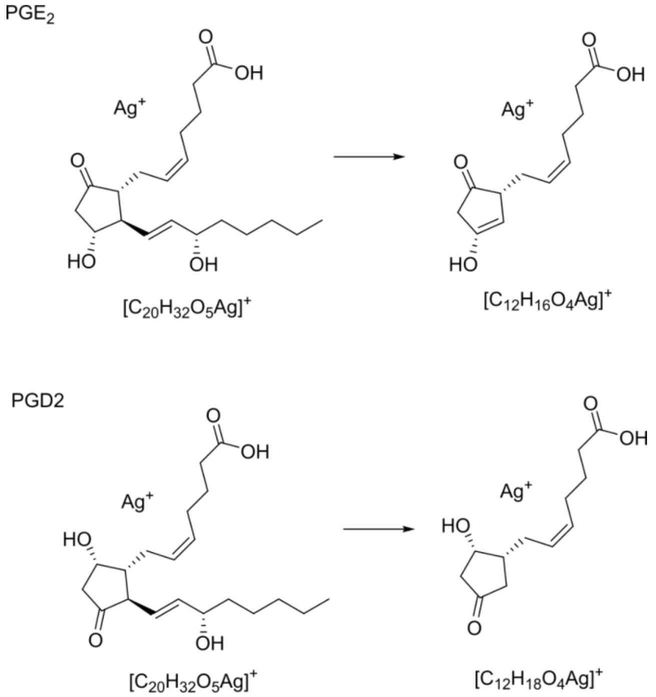

Figure S6. Proposed fragmentation pathways for silver adducts of PGE_2 and PGD_2 giving rise to product ions with m/z 331.0095 ($C_{12}H_{16}O_4Ag^+$) and 333.0252 ($C_{12}H_{18}O_4Ag^+$), respectively. The carbon chain losses of silver-cationized PGE_2 and PGD2 both arise from cleavage of the C8-C9 bond (Figure 1). Despite having the same site for fragmentation, the PGE_2 loses one more hydrogen (loss $C_8H_{16}O$) compared to PGD_2 (loss $C_8H_{14}O$). This difference is related to the adjacent carbonyl or hydroxyl groups present next to the cleaved bond. This indicates that the higher electronegativity of the carbonyl group compared to the hydroxyl group facilitates a fragmentation mechanism where the hydrogen atom of C8 is transferred to the C9 upon cleavage of the C8-C9 bond in the PGD_2. Interestingly, the cleavage of C8-C9 was observed in the MS^2 of PGD_2 yielding the product ion with m/z 333.0252 and in the MS^3 of PGE_2 giving rise to the product ion with m/z 331.0096 (Figure 1 and Table 1).

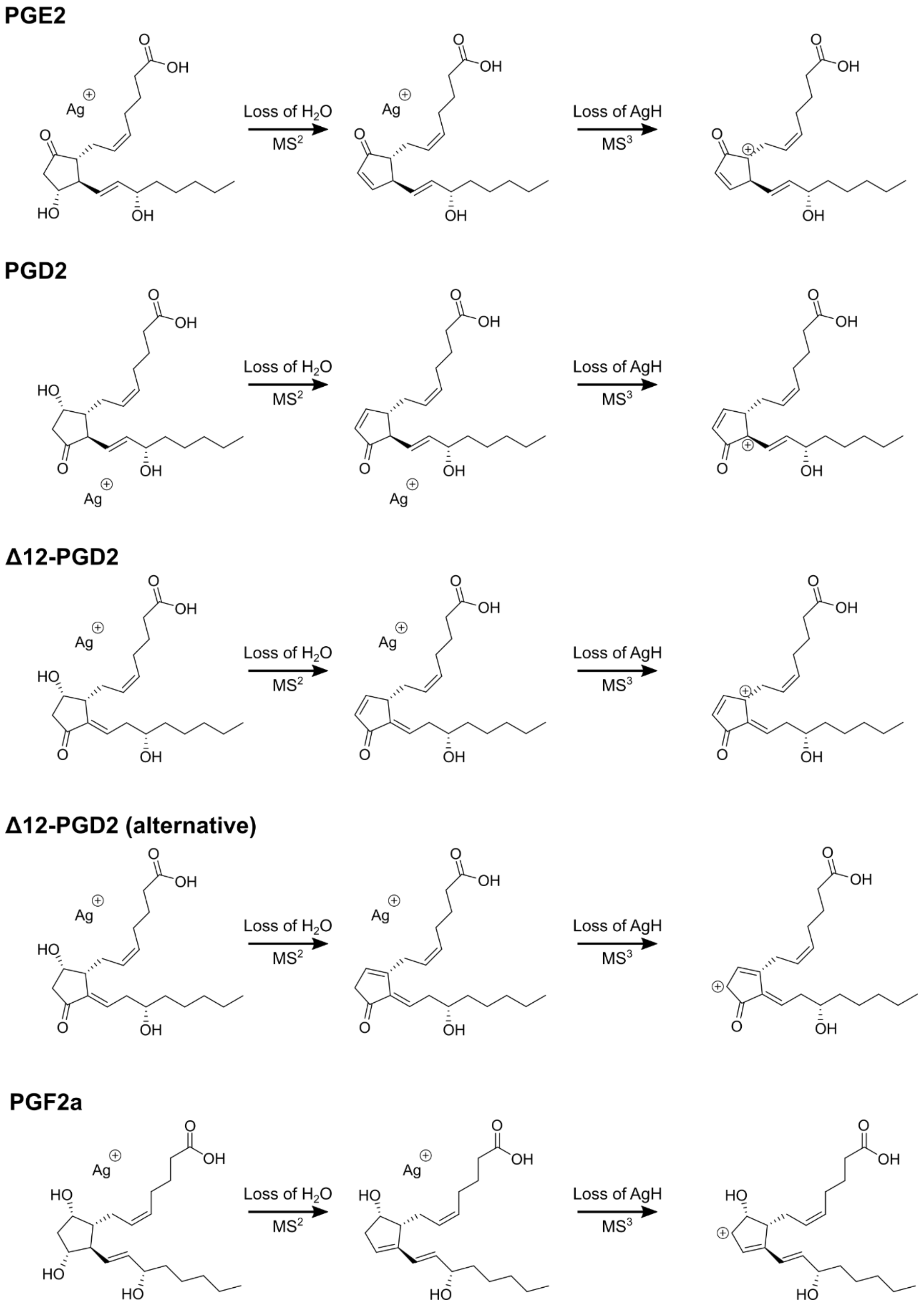

Figure S7. Proposed fragmentation pathways for PGE_2, PGD_2, Δ12-PGD_2 and PGF_{2a} when subjected to CID as silver adducts.

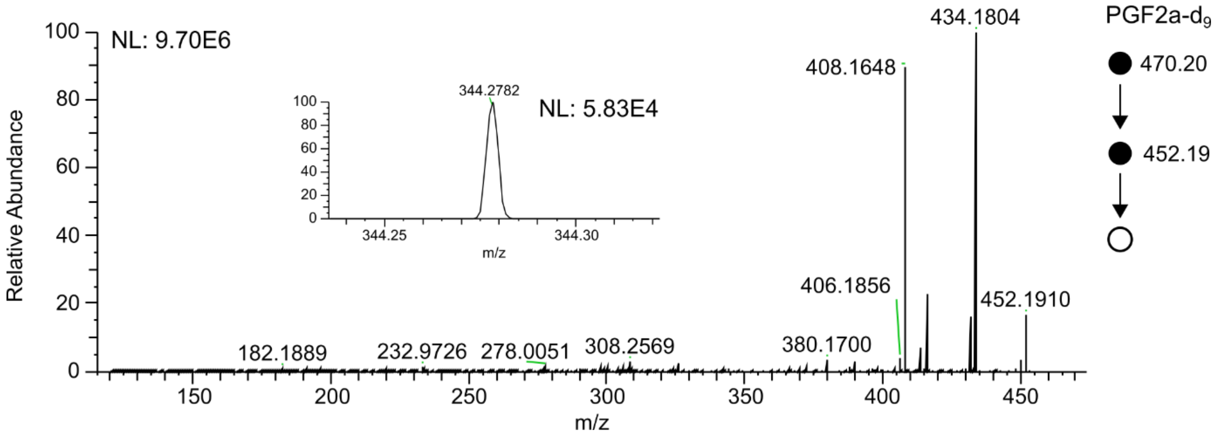

Figure S8. MS^3 spectrum of PGF_{2a}-d_9 detected as silver adduct at m/z 470.20. The loss of water at m/z 452.19 was isolated for MS^3. The insert shows the product ion after loss of AgH from the precursor 452.19.

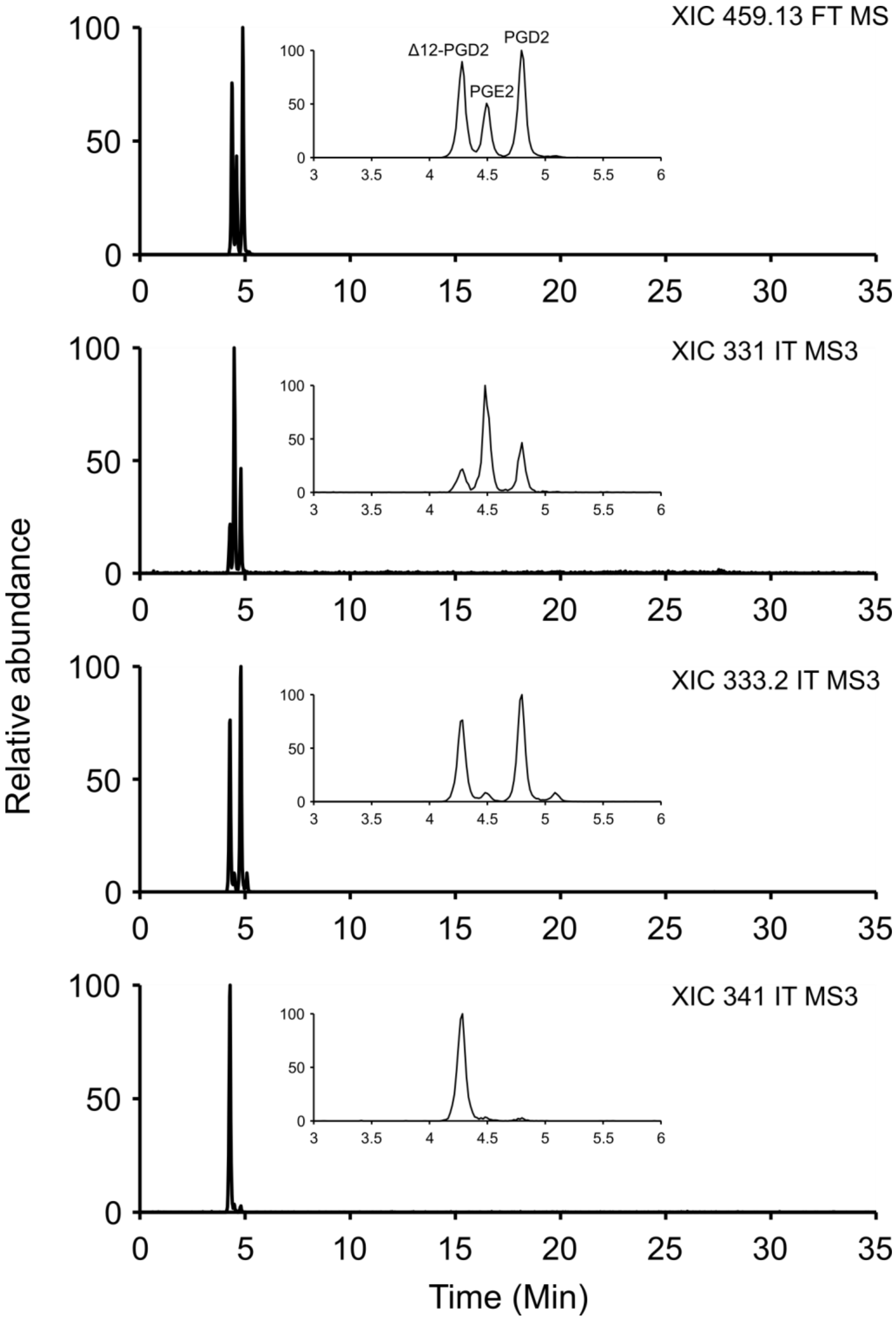

Figure S9. LC-MS analysis of PG isomers (PGE_2, PGD_2, Δ12-PGD_2) spiked (1 μM each) in rat brain extract. Shown are the XIC for m/z 459.13 (± 5 ppm) from the full scan FT-MS and XICs for the product ions m/z 331, 333.2 and 341 (± 0.5 amu) from the MS^3 spectra acquired in the ion trap. The elution order of the isomers shown in the top panel was determined by the corresponding MS^3 spectra when compared with the spectra of authentic standards. The small peak at the XIC of m/z 333.2 at 5.2 min originates from a low abundance degradation product in the standards of PGD_2 and Δ12-PGD_2 due to long-term storage of the diluted solutions in acetonitrile. The separation was achieved using the gradient shown in Table S4.

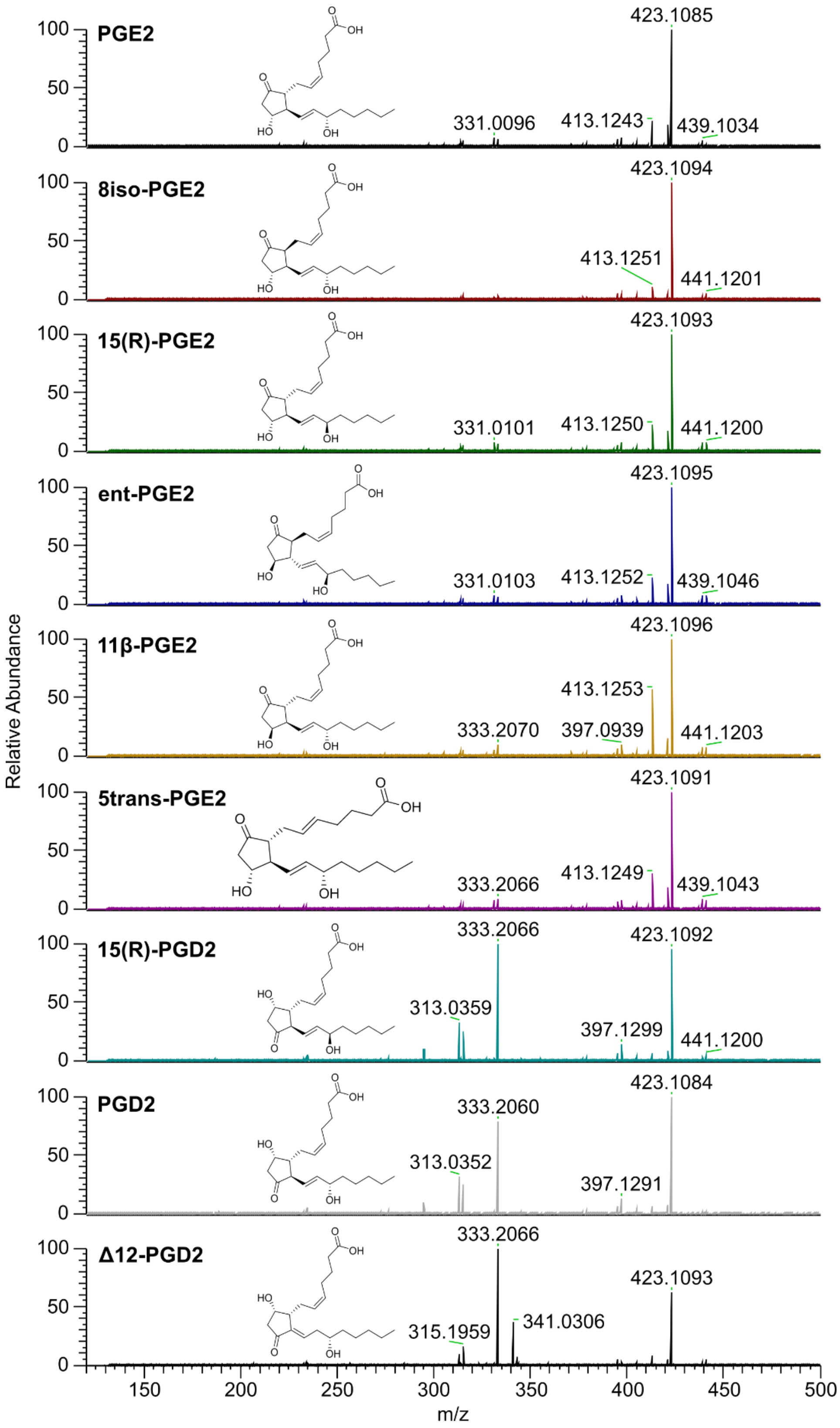

Figure S10. MS^3 spectra of various prostaglandin isomers. All spectra were acquired for the transition 459.13 -> 441.12 at CID 30 using an Orbitrap Velos Pro mass spectrometer with direct infusion (5 μL min^{-1}).

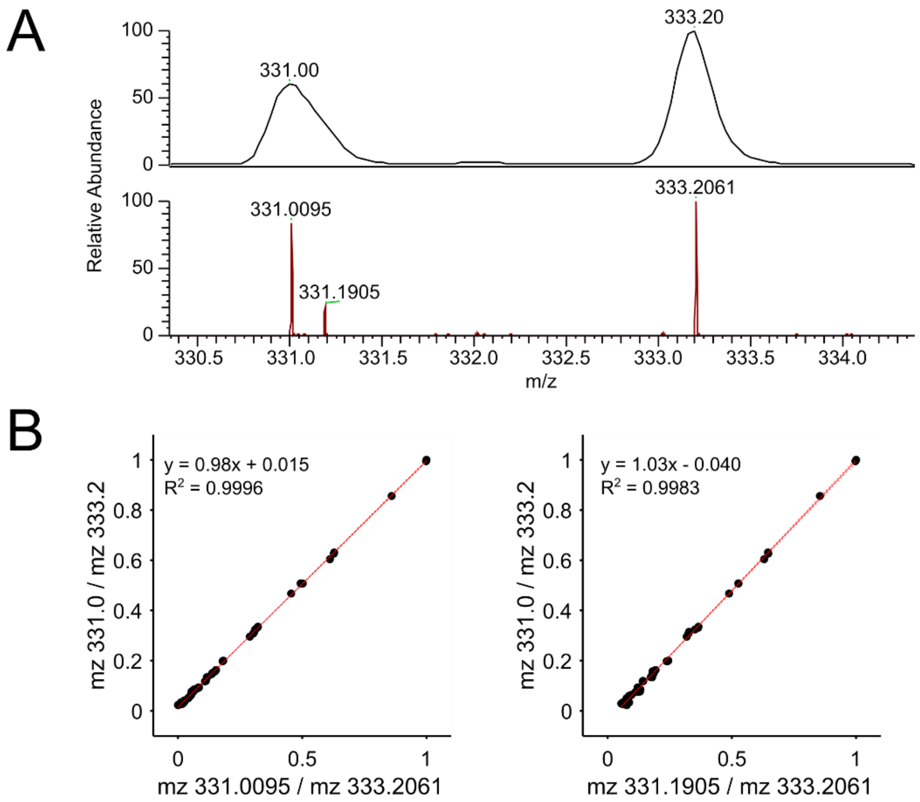

Figure S11. Comparison of FT and IT data in MS^3. A) IT and FT (60000 resolution) data from the analysis of PGE_2 standard showing that the MS^3 product ion (precursor: 459.13, CID fragmentation) with m/z 331 in IT contains two peaks in the FT. B) Ratio of m/z 331.0095 or 331.1905 to m/z 333.2061 in the FT compared to the ratio of m/z 331.0 / 333.2 in the IT, showing that the two comparisons correlate very well, thus, the presence of two peaks in the FT MS3 spectra does not affect the IT MS^3 data.

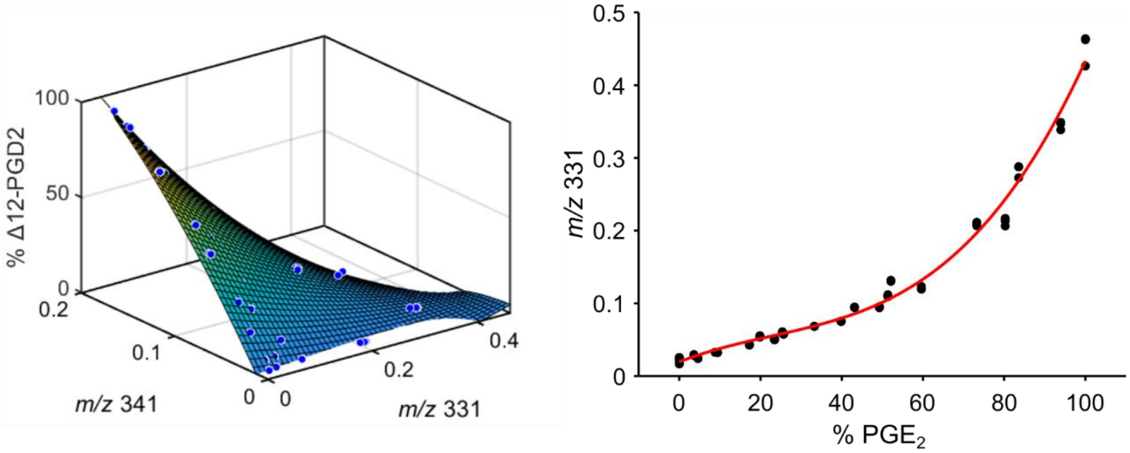

Figure S12. Prediction models for Δ12-PGD_2 (left) and PGE_2 (right) after analysis of solutions with various proportions of PGE_2, PGD_2 and Δ12-PGD_2. See main text for details on the fitted plots.

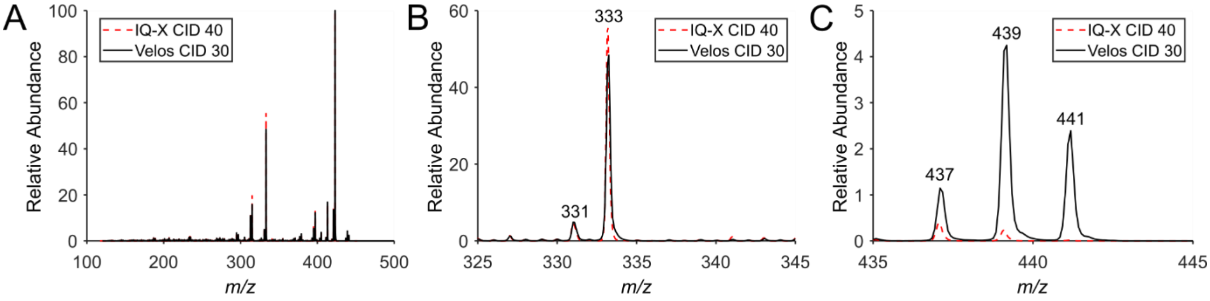

Figure S13. Dependence of product ions' abundance on CID level. A) MS^3 spectrum for the transition 459.13 → 441.12 of a solution containing 50% PGE_2 – 50% PGD_2 analyzed using an IQ-X Orbitrap with CID level of 40 (red dashed line) or an Orbitrap Velos with CID level of 30. B) Relative abundance of product ions *m/z* 331 and 333 that are used in the model. The different abundance of *m/z* 333 among the two CID settings will affect the obtained data and thus the model. C) Relative abundance of precursor (*m/z* 441) showing lower fragmentation efficiency using lower CID level. The activation Q and activation time was constant in both experiments.

Filtering of MS^3 product ions intensity using S/N ratio. Since the intensities obtained from the MSI data for the product ions are much lower compared to those used for training the model, a S/N ratio threshold was established for keeping only good quality data. Initially, the raw ion image of product ion with m/z 333 is produced from the MS^3 data acquired in the ion trap (IT). This image is used as a marker for tissue and glass locations using a threshold of 30 intensity units (top row). The glass pixels area is important for establishing an area where the noise can be calculated. For each product ion (i.e. m/z 331, 333 and 341), the S/N is calculated in each spectrum (pixel-wise) as follows:

$$S/N = \frac{X - N_{avg}}{N_{sd}} \quad \text{(eq. S1)}$$

Where X is the intensity of the product ion in each spectrum, N_{avg} is the average intensity of the product ion in the noise region (i.e. the glass pixels) and N_{sd} is the standard deviation of the intensity of the product ion in the same noise region. The S/N is calculated for each of the product ions with m/z 331, 333 and 341 and the distribution of these values are shown in Fig. S14 (middle row). Here we have set a threshold of S/N > 5 to filter out pixels that would introduce erroneous predictions. Thus, the pixels that have been selected for each product ion are shown in Fig S14 (bottom row). In the pixels where the S/N of the product ion is below the set threshold, the intensity values are set to 0.

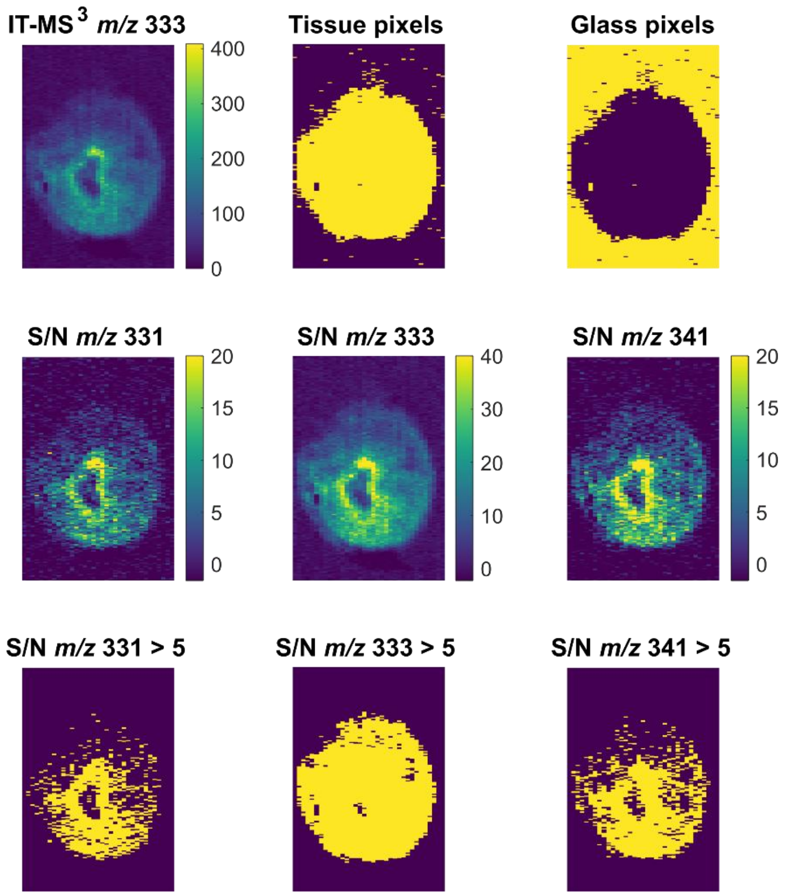

Figure S14. S/N filtering workflow for MSI data. Top row: Raw intensity image of product ion m/z 333 and selected pixels (yellow) that have intensity > 30 (tissue pixels) or intensity < 30 (“glass” pixels). Middle row: S/N distribution for each product ion using the equation S1. Bottom row: Selected pixels for each product ion with S/N > 5.

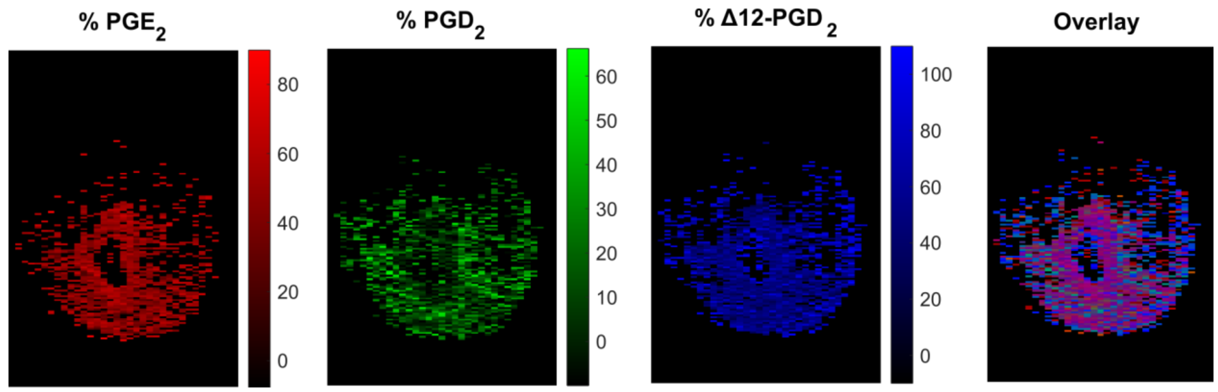

Figure S15. RGB overlay of the three isomer images of PGE_2, PGD_2 and $\Delta 12\text{-}PGD_2$. The individual images depict relative abundance of each isomer predicted by the model (see main text for details).

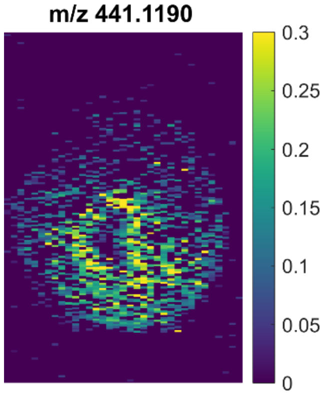

Figure S16. Ion image of m/z 441.119 annotated as Δ12-PGJ2 (accurate mass). The image is normalized to the signal intensity of the standard PGD_2-d_9.
